# Comparative strategic approaches to COVID-19 in Africa: Balancing public interest with civil liberties

**Published:** 2020-08-13

**Authors:** A E Obasa, S Singh, E Chivunze, T Burgess, F Masiye, T Mtande, J Ochieng, V Chalwe, B Mokgatla, S Rennie, K Moodley

**Affiliations:** 1 Centre for Medical Ethics and Law, Department of Medicine, Faculty of Medicine and Health Sciences, Stellenbosch University, Cape Town, South Africa; 2 Discipline of Dentistry, School of Health Sciences, University of KwaZulu-Natal, Durban, South Africa; 3 Division of Physiotherapy, Department of Health and Rehabilitation Sciences, Faculty of Health Sciences, University of Cape Town, South Africa; 4 Directorate of Research, Postgraduate Studies and Outreach, Malawi University of Science and Technology, Ndata Farm, Thyolo, Malawi; 5 University of North Carolina Project, Tidziwe Centre, Lilongwe, Malawi; 6 School of Biomedical Sciences, College of Health Sciences, Makerere University, Kampala, Uganda; 7 National Health Research Authority, Ministry of Health, Zambia; 8 International AIDS Vaccine Initiative, Botswana; 9 Department of Social Medicine, UNC Bioethics Center, University of North Carolina at Chapel Hill, USA

## Abstract

As COVID-19 spreads rapidly across Africa, causing havoc to economies and disruption to already fragile healthcare systems, it is becoming clear that despite standardised global health strategies, national and local government responses must be tailored to their individual settings. Some African countries have adopted stringent measures such as national lockdown, quarantine or isolation, in combination with good hand hygiene, mandatory wearing of masks and physical distancing, to prevent an impending healthcare crisis. The impact of stringent measures in low- to middle-income African countries has bought time for healthcare facilities to prepare for the onslaught of COVID-19 cases, but some measures have been challenging to implement. In some settings, public health measures have been associated with serious violations of individual rights owing to abuse of power and gaps in implementation of well-intentioned policy. Collateral damage with regard to non-COVID-19 diseases that were suboptimally managed in pre-pandemic times may mean that lives lost from other diseases could exceed those saved from COVID-19. While individuals complying with lockdown regulations have embraced an acceptance of the concept of the common good, at a broad community level many are finding the transition from individualism to collective thinking required during a pandemic difficult to navigate. In this article, we look at government responses to the pandemic in six African countries (Malawi, South Africa, Uganda, Zambia, Zimbabwe and Botswana), and highlight ethical concerns arising in these contexts.

Africa is currently in the grip of a global pandemic caused by SARS-CoV-2 infection. The resulting condition, COVID-19, has spread rapidly and infected over 4 million people globally.^[1]^ The pandemic has ravaged many nations on a global level, and African countries are no exception.

The World Health Organization (WHO) country and technical guideline for COVID-19 is subdivided into 16 different topics to inform countries about their preparedness for and response to the global pandemic. While these are important public health measures to contain an outbreak in more egalitarian contexts, some of the topics addressed in the guideline are tailored to a response in resource-rich settings.^[2]^ In Africa, given substantial inequities in health systems and socioeconomic conditions, our challenges vary, as under-privileged and vulnerable people are differently affected by the pandemic compared with those in resource-rich settings. In low- to middle-income countries (LMICs), some of the recommended critical measures for COVID-19 prevention and control could potentially and paradoxically be harmful, as they threaten survival. Fragile healthcare and public health systems render capacities for testing, isolating, quarantining, treating and contact tracing particularly difficult. In a pandemic, public health containment strategies must be balanced against realities such as lack of income, access to basic services and food security in different contexts.

Some African countries have implemented national lockdowns with stringent measures as a containment strategy. During the national lockdowns, temporary bans have been placed on both international and local travel, and citizens are encouraged to self-isolate and quarantine at their residences. South Africa (SA) has enforced compulsory wearing of face masks in public, and the country initially scaled up mass testing, community testing and contact tracing, at the centre of its multifaceted response. In public health emergencies, both isolation and quarantine are ethically justifiable. ^[3,4]^ An intrusion on the autonomy and privacy of individuals through contact tracing requires explicit ethical justification. The standard ethical justification is that the public health measures will reduce transmission and mortality and preserve healthcare capacity. Zambia and Uganda have taken a similar approach to SA; however, there is no evidence of massive community testing and mandatory wearing of face masks. The policy decisions made and implemented by governments in the region to date have inevitably varied. This article looks at these different responses to the COVID-19 pandemic in six African countries and highlights some of the ethical concerns that have arisen during the response in these countries.

## Malawi’s response to COVID-19

The President of Malawi formed a Cabinet Taskforce on COVID-19 comprising politicians from the ruling party with a mandate of managing the response to the outbreak before any confirmed positive case was reported in the country. Owing to irregularities, the committee was dissolved and a new Special Presidential Committee on COVID-19 comprising public health experts, cabinet ministers, civil society representatives, traditional leaders, religious leaders and opposition party representatives was formed at the end of April 2020. The first three COVID-19 cases in Malawi were reported on 3 April. Regional COVID-19 cases for Malawi, SA, Uganda, Zambia, Zimbabwe and Botswana (11 August) are shown in Table 1.

Strategies to control the spread of the COVID-19 pandemic have included the recruitment of 2 000 additional new health workers, establishment of nine laboratories across the country to conduct tests on suspected COVID-19 patients, community sensitisation messaging and surveillance, renovation of some wards in public health facilities to act as treatment centres for confirmed positive cases, and suspension of public gatherings of more than 100 people. As the number of cases increased, the Malawi Public Health Act of 1948 was updated to include strategies for managing the COVID-19 pandemic, including strict public health measures including social distancing, hand washing, wearing of face masks in public and a national lockdown, similar to other African countries. Although the national lockdown was planned to start on 18 April 2020, on 17 April the Human Rights Defenders Coalition obtained a court injunction to stop the process in a bid to protect the economy for the poor. Instead of a lockdown, the President announced an emergency cash transfer programme for the poor and small businesses.^[5]^

The first ethical concern in the Malawian context is the perceived restriction of individual autonomy. Although respect for persons and individual informed consent are fundamental ethical requirements for clinical care, in a pandemic, public health ethics based on the common good and public interest may override liberal individualism. ^[6]^ Balancing respect for persons with ensuring public safety has been challenging for public health experts and health workers. Both print and electronic media have reported that suspected cases of COVID-19 in Malawi are quarantined and tested without their explicit informed consent.^[6,7]^ This infringement of civil liberties is justified by law and the United Nations Siracusa Principles on the Limitation and Derogation Provisions in the International Covenant on Civil and Political Rights, provided that any limitation of rights is implemented humanely.^[7]^

There have been reported cases of stigmatisation and discrimination with regard to some confirmed cases of COVID-19. During updates on confirmed positive cases, there has been public disclosure of personal information and the public’s knowledge of the quarantine and treatment centres in their localities has made individuals who have tested positive to the virus identifiable, leading to stigmatisation of and discrimination against patients by community members.^[8,9]^ Health workers have also experienced stigma, to the extent that some were denied access to public transport because it was feared that they would spread the disease to fellow passengers.^[8,9]^ Healthcare workers face an ethical dilemma in balancing the principle of duty to care with their own safety owing to inadequate supplies of personal protective equipment (PPE). This conflict has led to industrial action to force government to provide PPE. It is recommended that both members of the Special Presidential Committee on COVID-19 and healthcare workers be on high alert for unethical practices. All stakeholders in the COVID-19 response must respect patients’ rights to informed consent, privacy and confidentiality to the fullest extent possible, provided the rights of others are not infringed, in keeping with the principles of public health ethics. The Special Presidential Committee must ensure that there is strong strategic community education and engagement to disseminate factual information on COVID-19 supported by scientific evidence.

## SA’s response to COVID-19

The first confirmed COVID-19 case in SA was reported in KwaZulu-Natal Province on 5 March 2020. This was characterised as an imported case (i.e. the infected person had travelled out of the country and contracted the disease). The strategy used by the SA government in response to COVID-19 is described in Box 1.

Cyril Ramaphosa, the South African President, declared a National State of Disaster on 15 March 2020 and thereafter announced the establishment of a National Command Council chaired by the President,^[10]^ which includes 19 cabinet ministers, the National Police Commissioner, the head of the South African National Defence Force, and a secretariat.^[11]^ On 25 March 2020, the country’s Ministerial Advisory Committee (MAC) on COVID-19 was officially established and published on 21 April 2020 by the National Department of Health.^[12]^ Regrettably, the MAC is orientated towards biomedical expertise, with the exclusion of social scientists, bioethicists, legal experts and community representatives.^[13,14]^

The implementation of a National State of Disaster led to the closure of schools and prohibition of gatherings of groups of more than 100 people. On 27 March 2020, a 21-day national lockdown was imposed, which was later extended by a further 2 weeks. Thereafter, on 1 April 2020, 67 mobile test units and 28 000 community healthcare workers were deployed. After 13 weeks at levels 5 and 4, national lockdown in response to the pandemic was relaxed to level 3 on 1 June 2020.

While the stringent level 5 lockdown measures were an accepted public health emergency response directed towards collective needs or the greater common good, they unavoidably limited individual health interests in a democratic society.^[15]^

The public health crisis also required re-prioritisation of health service delivery to COVID-19 patients. However, the health system also has an ethicolegal responsibility to provide uninterrupted services for the management of other health priorities such as HIV/AIDS and tuberculosis (TB), as well as contraceptive care, child immunisation, and support for individuals affected by gender-based violence (GBV).

Testing backlogs have been reported in SA. Although rigorous contact tracing is ideal, there is a need to balance this with available laboratory testing capacity; for effective diagnosis and treatment, a rapid turnaround time should be maintained to prevent unhealthy testing backlogs.^[4]^ Consequently, testing is now being reserved for hospitalised patients with suspected COVID-19 and healthcare workers.^[16]^ Based on reciprocal obligation to those who are saving lives while risking their own health, frontline healthcare workers are also prioritised for PPE and intensive care unit (ICU) admission. There are inherent limitations to self-isolation in low-income communities, but the potential removal of confirmed positive cases from communities, while necessary, may be stigmatising for positive cases and their families. The SA government issued arrests for non-adherence to self-isolation and quarantine requirements,^[17–19]^ which further stigmatised people infected with COVID-19. More collective effort is required from government, civil society, traditional leaders and other stakeholders to urgently address police brutality and GBV. Although these problems existed before the national lockdown, there have been increasing reports during lockdown.

Of note, lockdown measures up to 1 June included a temporary 9-week ban on the sale of alcohol. Together with reduced road traffic and physical distancing, this contributed to a significant reduction in motor vehicle accidents, trauma and hospital admissions.^[20]^ With level 3 lockdown, the alcohol ban was lifted and hospital wards in Western Cape and Gauteng provinces experienced an increase in alcohol-related trauma cases including GBV. This led to reinstatement of the alcohol ban on Sunday 12 July 2020.^[21]^

## Uganda’s response to COVID-19

Uganda adopted proactive public health measures such as voluntary self-isolation or quarantine and social distancing before confirmation of any COVID-19 cases. On 18 March 2020, more stringent interventions were implemented including closure of all schools and academic institutions, suspension of public gatherings of more than 10 people, temporary border closure to non-Ugandan citizens and mandatory quarantine for Ugandan citizens returning from high-risk countries. Factories, hotels, large plantations, markets and public transport were to operate while following standard operating procedures issued by the Ministry of Health. Later, the international airport and all other border points of entry were closed except for cargo aeroplanes and trucks.

Confirmation of the first case on 22 March 2020 triggered banning of public transportation, and later private transport, and closure of non-essential shops. Only essential services were allowed to operate with permits. These restrictions raised a number of issues, including inability of patients with non-COVID-19 conditions such as HIV, diabetes, hypertension or pregnancy, and healthcare workers, to travel to hospitals. After due consideration, patients and essential services personnel were later granted permission to secure permits, although only those who would afford private transport were able to benefit, leaving the poor who rely on public transport helpless, as the country has no ambulance system. The right to access healthcare in general was therefore seriously restricted by the focus on COVID-19. Although limitations of personal liberties may be justified during response to such pandemics, collateral harm to patients with other health conditions should not be ignored as occurred in Uganda, where no clear ethical framework or guidelines were used to manage or mitigate the ethical issues arising from COVID-19 public health interventions.^[22]^

There are also reports of human rights violations involving 19 youth living at a shelter designated for lesbian, gay, bisexual, transgender and intersex people in Kampala. They were allegedly arrested, physically assaulted and questioned on their sexual behaviour. Furthermore, they were charged for violating physical distancing rules and other COVID-19-related regulations; however, they were not detained under Uganda’s anti-homosexuality law. After intense pressure from human rights groups, charges were dropped, but only after 50 days of detention. Some of those detainees were HIV patients and were deprived of their antiretroviral drugs during the detention.^[23]^

As the number of cases of COVID-19 increases in Uganda, concern exists over refugee populations where physical distancing is not possible. Uganda hosts the largest number of refugees in Africa. Although there were no reported COVID-19 deaths at the time of writing, rapid spread of disease in refugee camps could have disastrous consequences.^[24]^

## Zambia’s response to COVID-19

According to the Public Health Act Cap 295 of the Laws of Zambia, the Minister of Health signed and introduced two statutory bills that designated COVID-19 a public health emergency and provided additional regulations to facilitate management and control of the disease. This bill was announced in the ministerial statement of 14 March 2020.

The Minister of Health encouraged all Zambian citizens to maintain a high level of hand hygiene and comply with the abovementioned measures. Health inspectors and authorised officers were deployed to enforce full compliance with the regulations. Citizens who failed to comply could be fined and held liable to penalties, as stipulated in the regulations. Furthermore, Zambia took steps to close schools and universities and prohibit church gatherings. The country also recruited over 2 000 health workers to enhance the fight against COVID-19, with ongoing surveillance activities and targeted testing.

The President of the Republic addressed the nation twice, on 25 March 2020 and later on 9 April 2020. In both addresses, he reiterated his obligation to protect Zambian citizens through the pandemic and encouraged full compliance. In a public health emergency, implementation of containment strategies must be balanced with their adverse economic impact. The infringement of freedom of movement and the call to work from home have resulted in economic hardship, with loss of revenue to citizens employed in both formal and informal sectors. There is a section of the population struggling economically. Overall, citizens feel that their individual rights of movement and access to a decent life are being violated. Zambia is an LMIC with limited healthcare facilities, and rapid spread of the virus may potentially devastate the healthcare system. Given that developed countries have struggled with ICU beds and ventilators, how will African countries cope?^[25]^ Currently in Zambia some of the ethical questions relate to who will get access to ventilators and who will be cared for in hospitals. What are the selection criteria? Does Zambia have enough ICU beds to care for citizens should the need arise? These are important ethical questions that need to be addressed before the country reaches its infection peak. The guidelines should be developed in such a way that healthcare resources, ventilators and ICU beds are not allocated to affluent families and friends of politicians should the need arise. To prevent this, a resource allocation committee would need to develop guidelines to communicate, and justify their rationale clearly and transparently.^[26]^ While other resource-constrained countries such as SA have clear triage guidelines, Zambia still needs to work on ensuring fair distribution of limited resources. In developing these key guidelines, trust, transparency and accountability should be central to answering questions around prioritisation and fair allocation of limited resources.

## Zimbabwe’s response to COVID-19

Zimbabwe is a landlocked country currently experiencing a myriad of political and economic challenges. It recorded its first confirmed case of COVID-19 on 21 March 2020. Prior to this, travellers had been subjected to screening at the three international airports since January 2020. Travellers were asked to share contact details for continued tracing purposes. Travellers from high-risk countries were also advised to self-isolate in line with WHO guidelines, while at the same time some civil liberties were denied. Few people, including those in positions of influence, followed these guidelines.^[27]^

The government rose into action after the death of the second case – a prominent young journalist and son of an influential top Zimbabwean politician and businessman, who had recently returned from New York, USA.^[28,29]^ His death received considerable media attention and raised concerns around the readiness of the government to respond to a pandemic that has ravaged the globe.

The Zimbabwe government reserved 425 hospital beds and 5 ventilators for COVID-19 patients in a tertiary care hospital in Harare, the capital, and also started upgrading all infectious disease centres across the country.^[30]^ At one such centre, located in Harare, 5 additional ventilators were installed. The government further repurposed 5 private hospitals as COVID-19 centres with capacity of approximately 650 beds and 20 ventilators. Four of these private hospitals are located in Harare and the fifth in Bulawayo, the second-largest city. Allocating resources to COVID-19 patients has reduced capacity for non-COVID-19 patients in a country with high prevalences of TB and typhoid. In light of these, there is a need to balance the COVID-19 response with the current pandemic response.^[31]^

The President of Zimbabwe declared COVID-19 a national disaster on 27 March 2020, which mapped out government policy issues during a public health emergency. Temporary bans were placed to prevent public gatherings of more than 50 people, schools were closed, and sporting events were cancelled. Public areas such as beer halls and swimming pools were closed, and religious gatherings were banned. Visits to hospital patients were restricted to one person once a day. These infringements on liberties were justifiable in response to a public health emergency. However, as indicated below, in the Zimbabwean context stringent measures threaten to have worse consequences than the COVID-19 pandemic for poor communities and neighbourhoods.

A national 21-day lockdown followed on 30 March that closed all facilities except for essential services such as public healthcare facilities and grocery stores. Allegations of heavy-handedness on the part of the police and the military have undermined the government’s effort.^[32]^ In certain instances, accessing healthcare facilities and other essential services was difficult as the police denied citizens entry at security checkpoints. The courts were also closed, and prison visits were suspended. These measures were necessary to contain infection spread, albeit with various ethical implications for justice and urgent legal processes.

Clearly, accountability and consistency are required from all citizens, including those in influential positions, who should lead by example and not defy announced measures to contain the spread of an infectious disease. The administrative structures in Zimbabwe allow for reach to household level through the political routes in the cities and traditional leader channels in rural areas. These can be used during the ongoing COVID-19 outbreak to educate the public and disseminate information. Allegations of security forces’ heavy-handedness may be avoided with proper education of citizens and improvement in training of security forces to highlight the avoidance of human rights abuses while implementing crisis management.

## Botswana’s response to COVID-19

Botswana, a landlocked country in the heart of southern Africa, with a population of just over 2.3 million people, has one of the strongest economies in the region and is politically stable.

Currently, Botswana has highly stringent protective measures in place. Initially, a 28-day total lockdown was implemented in order to mitigate the spread of COVID-19. Before the end of the initial lockdown period, a 6-month state of emergency was declared, granting the head of state the power to solely lift some of the lockdown rules or add to them. With schools and businesses closed, there was also cancellation of all social activities and mandatory government quarantine of all people arriving in Botswana or those with suspected exposure. The latter regulation saw the entire parliament of Botswana being subjected to a 14-day supervised quarantine similar to institutionalisation.

The government of Botswana introduced ‘movement permits’, required for anyone to leave home even to obtain essential items such as food. However, the permit system does not work well, as it is often unclear about what is permitted and what is not. Recently, as Botswana now plans to ease the lockdown, different permits have been introduced, e.g. a pink permit that employers can apply for to enable employees who were in a different city or village to be able to travel for work. This permit is valid for several days. A permit to be able to go to work is valid for 5 days, and one would then need to apply for renewal. The permit to go to supermarkets is valid for 4 hours only.

All travellers arriving in Botswana are subjected to a 14-day supervised quarantine and mandatory testing. Whenever a new COVID-19-positive case is announced, some details are included, such as gender, age, nationality and travel history. There have also been requests from members of the public via social media for the identity of individuals who tested positive to be disclosed. These details have the potential to compromise confidentiality. Consequently, there is growing concern about stigmatisation of and discrimination against those who have recovered from COVID-19 as they are integrated back into society.^[33–35]^

All COVID-19 patients are entitled to respect for their rights to privacy and confidentiality. Disclosing patients’ details that could easily be used to identify them has led to stipulations and circulations of pictures of suspected patients on social media. The Siracusa Principles were adopted by the United Nations Economic and Social Council in 1985 and are now firmly enshrined in international human rights law and standards. In a public health crisis, there are grounds for limiting certain human rights to permit the state to deal with a serious pandemic such as COVID-19; however, these measures must be specifically aimed at preventing the disease from spreading with minimum and necessary infringement of human rights.^[7]^ In this light, the sharing of pictures and identities of suspected COVID-19 patients, compromising patient privacy and confidentiality, is a direct violation of the Siracusa Principles. Furthermore, there is a growing risk of potential stigmatisation of and discrimination against suspected COVID-19 patients and their families. To address the latter, it is important that key strategic and consistent communication is packaged in such a way that it is educational and improves community understanding of why certain measures are in place. Communication of the rationale for public protection is even more important when strict public health measures are introduced.

## Discussion

The COVID-19 pandemic presents a major challenge to all countries across the globe. To curb the spread of the disease and reduce the mortality rate, each country relies on its government response and implementations thereof. With the exception of New Zealand,^[36]^ since the pandemic emerged, it has caused a high mortality rate in many resource-rich countries, albeit through poor leadership, uninformed scientific responses and constant denial such as occurred in the USA and the UK.

At the time of writing, all countries in Africa had registered positive cases of COVID-19 (except the tiny landlocked kingdom of Lesotho). Countries such as Uganda have recorded a low mortality rate, and SA has recorded an impressive recovery rate (~57%). Owing to the fragility of healthcare systems on the continent, African governments have had to take extremely difficult decisions to adopt draconian measures such as total lockdowns with stay-at-home orders. Governments and leaders have been forced to choose between saving lives and livelihoods. At the early stages of the pandemic, Botswana and SA implemented a hard lockdown; however, Malawi’s lockdown rules were challenged, and a court injunction was obtained to stop the national lockdown. SA introduced one of the world’s most restrictive COVID-19 lockdowns – including a temporary ban on alcohol and cigarette sales – but has been easing restrictions gradually and is currently on the third of five levels. Although the Constitutional Court found some of the lockdown regulations ‘invalid’ and ‘unconstitutional’, the lockdown rules still stand because the court suspended its declaration for a period of 14 days. During the lockdown, SA and Zimbabwe have experienced unethical police brutality that undermines the human rights and public health response.^[37]^ SA’s High Court declared an order to end police brutality during the national lockdown following the death of Collins Khosa. Zimbabwe is no stranger to police brutality with documented evidence. The Zimbabwe Human Rights Association (ZimRights) raised concerns over the increasing trend of police brutality during the lockdown under the guise of enforcing the directives of President Emmerson Mnangagwa.^[38]^

Sub-Saharan African countries have poor public healthcare systems and are burdened with communicable and non-communicable diseases. Allocation of resources to COVID-19 patients has reduced capacity for non-COVID-19 patients in LMICs with high prevalences of TB, diabetes and HIV. For instance, HIV is more prevalent in sub-Saharan African countries than in the rest of the world, and restriction on freedom of movement by curfews should be carefully balanced so as not to deprive non-COVID-19 patients in need of care and essential medication. In the short term, the current attention given to COVID-19 is disproportionate, and neglecting other diseases (both communicable and non-communicable) could pose a significant danger to the public health system in the long term. Prioritisation of care resources such as ICU beds and ventilators for COVID-19 patients has also raised significant ethical concern in countries such as Uganda and Zimbabwe. Uganda only had one ICU bed per million population^[39]^ at the start of the wave of COVID-19 infection, and Zimbabwe had about 25 ventilators.^[30]^ Owing to an overall lack of healthcare resources, a rapid surge in the rate of infections could lead to high mortality rates in both COVID-19 and non-COVID-19 patients.

Africa is currently experiencing a rapid surge in COVID-19 cases. In many African countries, bed capacities are limited. During the national lockdown, SA ramped up its bed capacity to prepare for the worst-case scenario.^[40]^ The provision of hospital beds solves part of the problem, but ‘hospital beds don’t cure patients – health care workers do’.^[40]^ Hospitals in Africa are understaffed, and lack appropriate resources compared with the Global North. In Malawi, healthcare workers are experiencing discrimination and there have been protests among health workers^[8]^ over salaries and lack of PPE.^[41]^ All these challenges have pointed to the need for African countries to improve their healthcare systems beyond the response to COVID-19. On a positive note, the early adoption of mandatory wearing of cloth masks has been implemented widely across the continent. Second, the use of high-flow nasal oxygen rather than invasive ventilation as treatment for critically ill COVID-19 patients has been shown to reduce mortality.^[42]^ Third, the most recent data on the beneficial effect of dexamethasone in severely ill patients, which reduced death rates by a third, could be a game-changer in Africa and beyond, as this steroid drug is relatively cheap and locally produced.^[43]^

## Conclusions

In the context of the COVID-19 pandemic, there is a need to balance individual autonomy with broader issues of public interest and safety. While public health measures have been taken in the best interests of communities^[44]^ in all six African countries described here, the ethics of implementation have been poorly communicated, poorly understood, and in some cases compromised. The impact of public health measures during COVID-19 in Africa is likely to be broad and long-lasting. However, this is not to suggest that these public health measures are unnecessary. The social, political, economic and psychological effects of allowing a pandemic to spread without implementing such public health measures are seemingly more devastating. The challenge arises when highly restrictive measures are superimposed on historical socioeconomic inequity.

We recommend that African government responses to COVID-19 should be contextualised and representative of the broad range of expertise necessary to assist with an outbreak that has strong ethical, legal and sociobehavioural components. Committees advising governments should incorporate scientists, bioethicists, legal experts and social scientists. In addition, all responses should have a strong ethics and human rights focus on community education and engagement of community healthcare workers, public health experts, civil society members, law enforcement officers and local community leaders.

## Figures and Tables

**Table 1.** Regional COVID-19 cases for Malawi, South Africa, Uganda Zambia, and Zimbabwe on 11 August 2020 (https://www.worldometers.info/coronavirus/)

	Total cases, *n*	Total deaths, *n*	Total recovered, *n*	Tests/1m population, *n*
Malawi	4 674	146	2 430	1 818
South Africa	563 598	10 621	417 200	55 018
Uganda	1 313	9	1 138	6 566
Zambia	8 275	241	7 004	5 143
Zimbabwe	4 748	104	64	3 561

## Box 1. South African government’s response to COVID-19

### Stage 1: Preparation

- Community education
- Establishing laboratory capacity
- Surveillance

### Stage 2: Primary prevention

- Social distancing and hand washing
- Closing schools and reduced gatherings
- Closure of borders to international travel

### Stage 3: Lockdown

- Intensifying curtailment of human interaction

### Stage 4: Surveillance and active case findings

Source: National Department of Health, 2020.
